# Captive Green Iguana Carries Diarrheagenic *Escherichia coli* Pathotypes

**DOI:** 10.3389/fvets.2020.00099

**Published:** 2020-02-26

**Authors:** Gerardo Uriel Bautista-Trujillo, Federico Antonio Gutiérrez-Miceli, Leonel Mandujano-García, María Angela Oliva-Llaven, Carlos Ibarra-Martínez, Paula Mendoza-Nazar, Benigno Ruiz-Sesma, Carlos Tejeda-Cruz, Liset Candelaria Pérez-Vázquez, Jesús Eduardo Pérez-Batrez, Jorge E. Vidal, Javier Gutiérrez-Jiménez

**Affiliations:** ^1^Facultad de Medicina Veterinaria y Zootecnia, Universidad Autónoma de Chiapas, Tuxtla Gutiérrez, Mexico; ^2^Instituto Tecnológico de Tuxtla Gutiérrez, Tuxtla Gutiérrez, Mexico; ^3^Instituto de Ciencias Biológicas, Universidad de Ciencias y Artes de Chiapas, Tuxtla Gutiérrez, Mexico; ^4^Rollins School of Public Health, Emory University, Atlanta, GA, United States; ^5^Department of Microbiology and Immunology, University of Mississippi Medical Center, Jackson, MS, United States

**Keywords:** diarrheagenic *Escherichia coli*, prevalence, Iguana, Chiapas, antibiotic resistance, zoonosis

## Abstract

The green iguana appears to be a carrier for bacteria causing gastrointestinal infections in humans. The presence of diarrheagenic *E. coli* (DEC) pathotypes, however, has not been studied in this reptile. The aim of the current work was to investigate the prevalence of DEC in the intestines of 240 captive green iguanas, their phylogenetic groups, and the antibiotic susceptibility profile. *E. coli* strains were isolated from 41.7% (*N* = 100/240) of the intestinal content of green iguanas. DEC strains was identified in 25.9% of the screened population and were detected in the majority (62%, *p* = 0.009) of those reptiles carrying *E. coli* strains. Among DEC strains, STEC strains carrying the *stx1* gene were the most prevalent pathotype isolated (38.7%), followed by EAEC and ETEC (27.4% each). Genetic markers of DEC strains belonging to the EHEC pathotype were not detected. More than a half of DEC strains were classified into the Clade I-II phylogroup (64.5%), followed by the phylogroup A (14.5%). The antibiotic susceptibility method demonstrated that a high proportion of DEC strains were resistance, or non-susceptible, to carbenicillin, amikacin, and ampicillin. We conclude that the green iguana kept in captivity is a carrier of DEC strains bearing resistance to first-line antibiotics, including penicillins. Given the increase presence of the green iguana in Latin American households, these reptiles represent a potential source of transmission to susceptible humans and therefore a potential source of gastrointestinal disease.

## Introduction

*Escherichia coli* is a commensal bacteria found in the gastrointestinal tract of human and animals. Its ability to acquire virulence genes has originated strains that cause serious gastrointestinal infections in humans as well as animals (1). *E. coli* pathogenic strains are responsible for ~56 million cases of diarrhea causing 0.2 million annual deaths worldwide, most of it in children between 2 and 5 years of age (2).

Based on the virulence traits of this Gram-negative bacterium and the location of the infection within the human host, pathogenic *E. coli* strains are classified in Diarrheagenic *E. coli* (DEC) and Extra intestinal *Escherichia coli* (ExPEC). This last group includes uropathogenic *E. coli* (UPEC) and neonatal meningitis *E. coli* (NMEC). The DEC group includes Enterotoxigenic *E. coli* (ETEC), Enteropathogenic *E. coli* (EPEC), Enteroinvasive *E. coli* (EIEC), Shiga toxin-producing *E. coli* (STEC), Enteroagregative *E. coli* (EAEC), and diffusely adherent *E. coli* (DAEC) (3). EHEC (Enterohemorrhagic *E. coli*) is a subgroup of STEC strains associated with outbreaks and severe clinical illness in humans (4). Among the DEC pathotypes, STEC infections cause diarrhea, and hemorrhagic colitis but the infection occasionally progress to hemolytic uremic syndrome (HUS) that despite severe sequela can cause death (5). STEC virulence is mainly based on its ability to produce two isoforms of the Shiga toxin (Stx): Stx1 and Stx2, related to infections in human, mainly subtypes Stx2 (6). Multiple STEC serotypes have been reported in outbreaks of human disease, being the most frequent STEC O157, followed by non-O157 serotypes including O26, O103, O111, O21, O45, and O145 strains (7).

It have been reported that bovines, ovines, goats, pigs, dogs, and cats are reservoirs of different DEC strains (8). Ruminants, for example, are an important reservoir of DEC strains with their feces representing a key source of contamination of water and food that cause infections to humans. Several epidemiological studies have demonstrated the zoonotic potential of STEC strains. For instance, a study conducted in Belgium analyzing diarrheic calves found that 58% of STEC strains were capable of inducing the attaching/effacement (A/E) lesion, followed by EPEC strains (38%); the O26 and O111 serogroups were the most frequent (47.5 and 30%, respectively) (9). Another study found a prevalence of 0.7% of STEC serotype O103:H2 strains in feces of Norwegian sheep (10). A work conducted in Iran showed the presence of STEC strains in healthy calves and goats (26.3 and 27.5%, respectively) (6).

Recently, in Mexico, a high prevalence of STEC (40.7%) and ETEC (26.7%) was observed in dairy cows, besides reporting the presence of *E. coli* serotype O157:H7 and O104:H12 (11). The presence of DEC strains in wild animals seems to be low in comparison to their prevalence in humans and ruminants. EPEC (typical and atypical) and STEC strains have been isolated from captive wild birds in Brazil (12, 13). A study that surveyed animals from zoos in India demonstrated a low prevalence of DEC strains in wild ruminants (STEC 7.14%; EPEC 1.58%), in non-ruminant animals (STEC 3.48%; EPEC 5.81%) and wild birds (EPEC 5.84%) (14).

In Mexico and in other South American countries, the green iguana (*Iguana iguana* Linnaeus, 1758) has played an important role in the economy of some regions of these Latin America countries as these animals are sold for human consumption but also kept as pets. These factors led to a dramatic decline of iguana populations the last few years. Important efforts, however, to preserve the species have emerged in Mexico such as a special protection is in place according to the Mexican Official Norm NOM-059-SEMARNAT-2010 (15) as well as efforts to promote their conservation by means of the Wildlife Management Units (WMU) (16).

Iguana species can carry in their intestines *Salmonella* species (17, 18) and *Escherichia coli* (19–21). The prevalence of *E. coli* in iguana species ranges from 40 to 70%. For example, in the Ricord's iguana (*Cyclura ricordi*) the prevalence reported was 50% (22), while in the land iguana (*Conolophus pallidus*) from the Galapagos Island it was 70.83% (19). Regarding the green iguana, studies have reported an *E. coli* prevalence of 40% (21) and 53.2% (18). To the best of our knowledge, whether the green iguana can carry DEC pathotypes has not been previously investigated and it was the main motivation for this study.

Therefore, the goal of this study was to investigate the prevalence of DEC strains colonizing the intestines of captive *I. iguana*. To figure out whether a subset of DEC strains is associated to intestinal colonization of the green iguana, we also determined their phylogenetic group as well as their susceptibility to first-line antibiotics.

## Materials and Methods

### Green Iguana Population

This study was approved by the Committee for Animal Care of the University Autonomous of the Chiapas state (approval ID number 06/VET/RPR/269/16). From autumn 2015 to spring 2017, a total of 240 captive *I. iguana* were selected from the WMU as follows: 112 specimens from the Istmo-Costa region and 128 iguanas from the Metropolitan region of Chiapas, Mexico. Those regions of the State of Chiapas concentrate the majority of WMU for green iguana. The reptiles at WMU from the Metropolitan region were maintained in cages of 27 or 253 ft^3^, while reptiles from the Istmo-Costa region were placed in wired mesh confinements of 16 × 33 ft. Lettuce, carrots, and cabbage constitute the daily food source and potable water was available *ad libitum* from water fountains. The reptiles were safely retrieved from their respective cages and were classified in accordance to their age: 126 juvenile iguanas (6–18 months of age) and 114 adults (more than 36 months of age). As juvenile iguanas present physical characteristics that do not allow distinguishing males from females, sex was determined only within the adult population. Male iguanas presented swelling of the hemipenis (females lack of this characteristic) and bigger femoral pores than females (23). The study utilized 55 females and 59 males.

### Isolation of *E. coli* and Identification of DEC Strains

Intestinal content was collected in accordance to approved guidelines (24). Briefly, a sterile swab was introduced into the cloaca and after rotating gently, it was placed in Stuart agar gel (Copan) for its transportation to the laboratory. Swabs were streaked immediately onto Eosin and Methylene Blue agar (BD-BBL) and incubated at 37°C for 24 h. Up to 10 lactose fermenting colonies were selected and analyzed by standard biochemical tests. MacConkey agar plates with sorbitol were used for the detection of EHEC serotype O157:H7 (25). *E. coli* strains were also genetically identified by PCR amplification of the *uidA* gene, that encodes for β-glucuronidase specific for *E. coli* (26).

### Diarrheagenic *E. coli* Pathotypes, Serogroups, and Phylogenetic Group's Identification by Polymerase Chain Reaction (PCR)

The following reference strains were used: *E. coli* strain (ATCC® 25922™) as a negative control, while EAEC 042 (044:H18), ETEC H10407 (O78:H11), EPEC E2348-69 (O127:H6), STEC EDL933 (O157:H7), and EIEC E11 (O124NM) prototype strains were used as a positive control in PCR reactions. Strains were kindly provided by Dr. Teresa Estrada-García from CINVESTAV, Mexico, and deposited into the bacterial collection of the University of Sciences and Arts of the state of Chiapas (27).

To obtain bacterial DNA, bacterial lysates of each of the 10 previously selected colonies were prepared by resuspending it in 1 mL of deionized water and then boiling the preparation for 10 min. The bacterial lysate was centrifuged at maximum speed for 5 min and DNA-containing supernatant was separated and stored at −80°C until used. Specific gene-targets for Enteroagregative *E. coli* (EAEC) (*aap, agg*R, and AAprobe genes) were amplified by a technique described by Cerna et al. (28). For Enterotoxigenic *E. coli* (ETEC) (*lt* and *st genes*), Enteropathogenic *E. coli* (EPEC) (*bfp*A and *eae*A genes), Shiga toxin-producing *E. coli* (STEC) (*stx*1 and *stx*2 genes), and Enteroinvasive *E. coli* (EIEC) (*ial gene*), we utilized targets and conditions described by López-Saucedo et al. (29). The *eae* and *hly*A genes, molecular markers for Enterohemorragic *E. coli* (EHEC), a well-known STEC sub-group that cause disease in humans, were amplified as established by Oswald et al. (30) and Schmidt et al. (31), respectively. The STEC strains were further analyzed in order to detect the O26, O45, O103, O111, O113, O121 and O145 antigens. To this end, primers amplifying the *wzx* gene (O- antigen flippase) in the O-antigen gene clusters were utilized as described by DebRoy et al. (32). The O157 antigen was investigated by amplifying the *rfb* gene (O- specific polysaccharide), according to Paton and Paton (33).

*E. coli* strains were further classified, using a quadruplex PCR method, into seven phylogroups: A, B1, B2, C, D, E, F, and the cryptic Clade I (34). This method is based in the presence or absence of *chuA, yjaA, arpA, trpA* genes and the TSPE4 C2 DNA fragment. PCR conditions and primers are shown in Table 1. PCR reactions were run in a thermal cycler C1000 (BioRad) and PCR products were analyzed through electrophoresis in agarose gel (2%) ran at 80 V for 1 h. Agarose gels were stained with SyberGreen® (Invitrogen), and photographed with the Molecular Imager® Gel Doc™ XR System (BioRad). The lambda molecular weight markers (100 and 1,000 bp) were used (Invitrogen, USA).

**Table 1:** Primers used in this study.

**Primer pair**	**Sequence (5^**′**^-3^**′**^)**	**Target**	**Encoded protein**	**Size (bp)**	**Reference**
uidA	AAAACGGCAAGAAAAAGCAG (F^*^) ACGCGTGGTTAACAGTCTTGCG (R^**^)	*uidA*	β-glucuronidase	147	(26)
aap	CTTGGGTATCAGCCTGAATG (F) AACCCATTCGGTTAGAGCAC (R)	*aap*	Aggregative adherence fimbria	310	(28)
aggR	CTAATTGTACAATCGATGTA (F) AGAGTCCATCTCTTTGATAAG (R)	*aggR*	Transcriptional activator	457	(28)
AA probe	CTGGCGAAAGACTGTATCAT (F) CAATGTATAGAAATCCGCTGTT (R)	AA probe	Anti-aggregation protein transporter	629	(28)
lt	GGCGACAGATTATACCGTGC (F) CGGTCTCTATATTCCCTGTT (R)	*lt*	Heat-labile enterotoxin	450	(29)
st	ATT TTTCTTTCTGTATTGTCTT (F) CACCCGGTACAAGCAGGATT (R)	*st*	Heat-stable enterotoxin	190	(29)
bfpA	AATGGTGCTTGCGCTTGCTGC (F) GCCGCTTTATCCAACCTGGTA (R)	*bfpA*	Bundle-forming pilus	324	(29)
eaeA	GACCCGGCACAAGCATAAGC (F) CCACCTGCAGCAACAAGAGG (R)	*eaeA*	Intimin	384	(29)
ial	GGTATGATGATGATGAGTCCA (F) GGAGGCCAACAATTATTTCC (R)	*ial*	Invasion-associated locus	650	(29)
stx1	CTGGATTTAATGTCGCATAGTG (F) AGAACGCCCACTGAGATCATC (R)	*stx1*	Shiga toxin 1	150	(29)
stx2	GGCACTGTCTGAAACTGCTCC (F) TCGCCAGTTATCTGACATTCTG (R)	*stx2*	Shiga toxin 2	255	(29)
SK1	CCCGAATTCGGCACAAGCATAAGC (F)	*eae*	Structural gene for intimin	881	(30)
SK2	CCCGGATCCGTCTCGCCAGTA TTCG (R)				
hlyA1	GGTGCAGCAGAAAAAGTTGTA G (F)	*hlyA*	α-hemolysin	1,550	(31)
hlyA4	TCTCGCCTGATAGTGTTTGGTA (R)				
O26	CAATGGGCGGAAATTTTAGA (F) ATAATTTTCTCTGCCGTCGC (R)	*wzx* (O26)	O-antigen-flippase	155	(32)
O45	TGC AGT AAC CTG CAC GGG CG	*wzx* (O45)	O-antigen-flippase	238	(32)
O45	AGCAGGCACAACAGCCACTACT				
O103	TTGGAGCGTTAACTGGACCT	*wzx* (O103)	O-antigen-flippase	321	(32)
O103	GCTCCCGAGCACGTATAAAG				
O111	TGTTTCTTCGATGTTGCGAG	*wzx* (O111)	O-antigen-flippase	438	(32)
O111	GCAAGGGACATAAGAAGCCA				
O113	TGCCATAATTCAGAGGGTGAC	*wzx* (O113)	O-antigen-flippase	514	(32)
O113	AACAAAGCTAATTGTGGCCG				
O121	TCCAACAATTGGTCGTGAAA	*wzx* (O121)	O-antigen-flippase	628	(32)
O121	AGAAAGTGTGAAATGCCCGT				
O145	TTCATTGTTTTGCTTGCTCG	*wzx* (O145)	O-antigen-flippase	750	(32)
O145	GGCAAGCTTTGGAAATGAAA				
O157	CGGACATCCATGTGATATGG (F) TTGCCTATGTACAGCTAATCC (R)	*rfbO157*	Polisacárido específico O	259	(33)
chuA.1b	ATGGTACCGGACGAACCAAC (F)	*chuA*	Membrane hemin receptor	288	(34)
chuA.2	TGCCGCCAGTACCAAAGACA (R)				
yja.1b	CAAACGTGAAGTGTCAGGAG (F)	*yjaA*	Stress response protein	211	(34)
yja.2b	AATGCGTTCCTCAACCTGTG (R)				
ArpAgpE.	GATTCCATCTTGTCAAAATATGCC (F) GAAAAGAAAAAGAATTCCCAAGAG (R)	*arpA*	Regulator of acetyl CoA synthetase	301	(34)
trpAgpC.1	AGTTTTATGCCCAGTGCGAG (F)	*trpA*	Operon leader peptide	219	(34)
trpAgpC.2	TCTGCGCCGGTCACGCCC (R)				
trpBA	CGGCGATAAAGACATCTTCAC (F) GCAACGCGGCCTGGCGGAAG (R)	*trpA* (control interno)	Operon leader peptide	489	(34)
TSPE4 C2	CACTATTCGTAAGGTCATCC (F) AGTTTATCGCTGCGGGTCGC (R)	TspE4.C2	Anonymous DNA fragment	152	(34)

* F, forward;

** *R, reverse*.

### Antimicrobial Susceptibility Analysis

The disk- diffusion method was utilized following recommendations by the Clinical and Laboratory Standards Institute (35). The following antimicrobial susceptibility test discs (BD BBL™ Sensi-Disc™) were utilized, according to different antimicrobial categories: Penicillins, ampicillin (AMP;10 μg) and carbenicillin (CAR;100 μg); aminoglycosides, amikacin (AMK;30 μg), netilmicin (NET;30 μg) and gentamicin (GEN;10 μg); cephalosporins, cephalothin (CEF;30 μg) and cefotaxime (CTX;30 μg); quinolones, ciprofloxacin (CIP;5 μg) and norfloxacin (NOR;10 μg); phenicols, chloramphenicol (CHL;30 μg); folate inhibitor, trimethoprim-sulfamethoxazole (SXT;25 μg) and furans, nitrofurantoin (NIT;300 μg). Strains resistant to β-lactams were further analyzed with amoxicillin-clavulanic acid discs (AMC; 20/10 μg). *E. coli* Non-susceptible strains (including intermediate and resistant phenotypes) to at least three antibiotics from different antimicrobial categories were recorded as multi-drug resistant (MDR) strains (36).

### Statistical Analysis

Prevalence of *E. coli* pathotypes, serogroups, phylogenetic groups, and susceptibility to antibiotics were analyzed by descriptive statistics. Proportions of typical molecular markers among DEC strains were analyzed by binomial test. The analysis between categorical variables was performed using the two-tailed Fisher's Exact test or Chi- Square test (when the frequencies were higher than 5) establishing a significance level when *p* < 0.05. All statistical analyses were conducted using the IBM SPSS statistics software (Chicago, SPSS Inc.).

## Results

### Diarrheagenic *E. coli* Identification

We isolated 100 *E. coli* strains of fecal samples collected from 240 iguanas kept in captivity. DEC strains was identified in 25.9% of the screened population of *I. iguana* and were detected in the majority [62% (*N* = 62), *p* = 0.009] of those reptiles carrying *E. coli* strains (*N* = 100). Among DEC strains, STEC (40.3%) was the most prevalent category followed by EAEC (27.4%, with a significative frequency of the *aap* gene) and ETEC (27.4%). EPEC strains had the lowest prevalence (4.9%) (Table 2). EHEC O157:H7 gene markers were not amplified by PCR neither EHEC strains were detected on MacConkey/sorbitol agar plates.

**Table 2:** DEC strains and their typical virulence genes isolated from *I. iguana* in Chiapas, Mexico.

**Feces samples with DEC pathotype: % (*N*)**	**Virulence gene: % (*N*)**	***p***
STEC: 40.3 (25)	*stx1:* 38.7 (24)	0.000002
	*stx2:* 1.6 (1)	
EAEC: 27.4 (17)	*aap:* 27.4 (17)	0.0001
	AA probe: 1.6 (1)	
ETEC: 27.4 (17)	*lt:* 19.3 (12)	0.359
	*st:* 11.3 (7)	
EPEC: 4.9 (3)	*bfpA:* 4.9 (3)	1.000
	*eaeA:* 3.2 (2)	
Total: 100.0 (62)		

Our PCR approach revealed a statistically significant higher frequency of STEC strains carrying the *stx1* gene in comparison to those carrying *stx2*. The *eae* and *hlyA* virulence genes were not amplified in any of these STEC strains suggesting that they did not belong to the EHEC category. Accordingly, O- antigen genes carried by EHEC's most common serotypes (e.g., O26, O45, O103, O111, O113, O121, O145, and O157) were not amplified in any of these strains.

### Investigation of Phylogenetic Groups

Given that these iguanas are in captivity we sought to investigate whether DEC strains were related by obtaining their phylogenetic group. Among these *E. coli* strains (*N* = 62), the clade I or II phylogroup was significantly the most predominant (64.5%), followed by phylogroups A and B2. Some of DEC strains (17.8%) could not be assigned to a known phylogroup (Table 3). Thus, DEC isolated from *I. iguana* may be related since the majority of strains were classified in clade I or II.

**Table 3:** Phylogenetic groups of DEC strains isolated in captive *I. iguana* in Chiapas.

		**DEC strains % (*****N*****)**					***p***
**Phylogenetic groups**	**STEC stx1**	**EAEC**	**ETEC**	**EPEC**	**STEC stx2**	**Total**	
A	25.0 (6)	0.0 (0)	17.6 (3)	0.0 (0)	0.0 (0)	14.5 (9)	0.005
B2	0.0 (0)	5.8 (1)	5.9 (1)	0.0 (0)	0.0 (0)	3.2 (2)	
Clade I-II	75 (18)	70.5 (12)	41.2 (7)	66.6 (2)	100.0 (1)	64.5 (40)	
Unknown	0.0 (0)	5.8 (4)	35.3 (6)	33.3 (1)	0.0 (0)	17.8 (11)	

There was not association between the prevalence of *E. coli* pathotypes and the age or sex of the iguanas. Regarding the geographic region were these reptiles are kept in captivity, a statistically significant association was observed between STEC strains isolated from iguanas kept at the Metropolitan (i.e., urban) region in comparison to those from the Istmo-Costa (i.e., rural) (Table 4).

**Table 4:** Associations among DEC strains and some characteristics of *I. iguana* in Chiapas, Mexico.

**Characteristic of *I. iguana* (N)**	**STEC stx1 % (*N*)**	**EAEC % (*N*)**	**ETEC % (*N*)**	**EPEC % (*N*)**	**STEC stx2 % (*N*)**	***p***
**Age**						
Juvenile (126)	10.3 (13)	8.7 (11)	5.5 (7)	2.4 (3)	0.0 (0)	0.337
Adult (114)	9.6 (11)	5.2 (6)	8.7 (10)	0.0 (0)	0.8 (1)	
**Sex**						
Male (59)	11.9 (7)	5.0 (3)	8.5 (5)	0.0 (0)	0.0 (0)	0.91
Female (55)	9.0 (5)	5.4 (3)	9.0 (5)	0.0 (0)	2.0 (1)	
**Region**						
Metropolitan (127)	18.9 (24)	9.4 (12)	7.1 (9)	0.0 (0)	0.8 (1)	<0.001
Istmo-Costa (113)	0.0 (0)	4.4 (5)	7.0 (8)	2.6 (3)	0.0 (0)	

### Antimicrobial Susceptibility of DEC Strains

**Figure 1:**
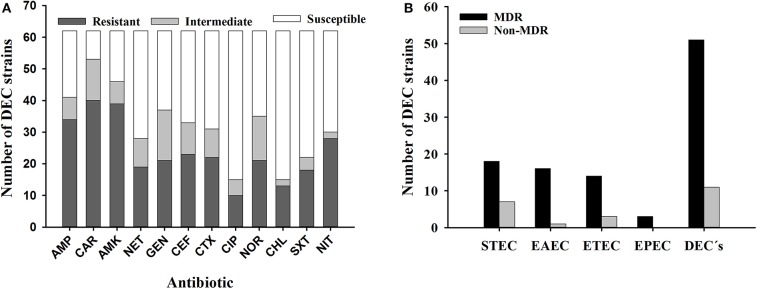
Antibiotic susceptibility of DEC strains (N = 62) isolated from *I. iguana* in Chiapas, Mexico. A, DEC strains with susceptibility, intermediate resistance or resistance against antibiotics. B, MDR phenotype among DEC strains. AMP; Ampicillin, AMK; Amikacin, CAR; Carbenicillin, GEN; Gentamicin, NET; Netilmicin, CEF; Cephalotin, CTX; Cefotaxime, CIP; Ciprofloxacin, NOR; Norfloxacin, CHL; Chloramphenicol, SXT; Trimethoprim-sulfamethoxazole, NIT; Nitrofurantoin; MDR, multidrug-resistant; STEC, Shiga toxin-producing E.coli; EAEC, Enteroaggregative E. coli; ETEC, Enterotoxigenic E. coli.; EPEC, Enteropathogenic E. coli; DEC, diarrheagenic E. coli.

Because these DEC strains represent a potential source of contamination for humans and therefore a source of gastrointestinal disease, we investigated their susceptibility to antibiotics utilized to treat gastrointestinal disease caused by Gram negative bacteria. More than a half of DEC strains were non-susceptible to antibiotics from the penicillin category, as well as two out of three antibiotics belonging to aminoglycosides. This trend was also observed when DEC strains were tested for their susceptibility to cephalothin and norfloxacin. 82.3% (*N* = 51) of DEC strains were non-susceptible to at least three antibiotics from different antimicrobial categories, and therefore strains were considered as multi-drug resistant (MDR). All strains non-susceptible to ampicillin were susceptible to amoxicillin-clavulanic acid (data not shown). In addition, the majority of strains were susceptible to ciprofloxacin and chloramphenicol (Figure 1).

## Discussion

*Escherichia coli* is an ubiquitous Gram negative bacteria considered as one of the main constituent of the gastrointestinal tract of animals (37), humans (38), as well as reptiles such as turtles (39) and snakes (40). Nevertheless, there is almost no information about the presence and prevalence of *E. coli* pathotypes, such as DECs in the intestines of reptiles (41). This study revealed that the majority of *E. coli* strains isolated from *I. iguana* carried genes encoding virulence factors associated with diarrhea in humans (62%) such as those carried by STEC, EAEC, or ETEC pathotypes. We found similar carriage prevalence of the pathogenic variants of DEC strains in *I. iguana* regardless sex or age.

How do Iguanas acquire pathogenic DEC strains? Contamination of their food and water sources seems to be the main driver for iguanas to acquire DEC strains. Preliminary results from our group found a high prevalence of DEC strains in water and food samples (52 and 48%, respectively) from the Metropolitan region than that in the Istmo-Costa region (17 and 13%, respectively); STEC strains was the more frequently pathotype isolated. Besides that water offered to iguanas in the Metropolitan region might be contaminated with DEC strains, cleaning of their water fountains can take up to a week allowing further growth of DEC strains in that water source. Given that cattle farms are not commonly found in the metropolitan region, and cattle have been reported as a main reservoir for DEC strains, the source of DEC strains that contaminate water in the urban sites might be human feces.

Several studies have demonstrated that *E. coli* pathotypes are isolated from dairy cows (8), sheep (42), and pigs (43). In Mexico, a prevalence of 40.7 and 26.7% for the carriage of STEC or ETEC in bovine, respectively, was reported in the states of Jalisco, Sinaloa, and Sonora (11). The prevalence of STEC strains among livestock in southern Mexico is unknown but current studies in our laboratories are investigating the presence of DECs in livestock in the same geographical regions as those studied in this work. To the best of our knowledge, however, the presence of DEC strains in the intestine of iguanas had not been reported.

Acquisition of these DEC strains also can be a result of coprophagy, a behavior seen in iguanas because it is required for biochemical digestion of vegetables of their diet (44). Captivity in urban settings along with the mentioned coprophagy should also account for the presence of DEC strains in the intestine of iguanas. These observations are supported by the fact that a statistical significant association was observed in this study between intestinal carriage of STEC strains and iguanas kept in captivity at a metropolitan region of Chiapas. Reptiles may be in closer contact with human feces because of the crowdedness of an urban setting. Data supporting this hypothesis includes our own observations reporting the isolation of DEC's (13.8%) from the feces of 94 children from Chiapa de Corzo, a locality belonging to the metropolitan region (45). Our findings agree with those by Sylvester et al. (21) whom demonstrated an association between the prevalence of *E. coli* carriage and certain geographic regions. We hypothesize that factors such as the weather, season of the year (summer and autumn), such as well as a potential hygiene deficiency when handling iguanas (fecal contamination in water and food in cages) favors the acquisition of DEC bacteria (46, 47).

In the current study, the prevalence of STEC carrying the *stx1* gene was higher than that of those carrying the *stx2* gene (Table 1). However, these strains were not EHEC as none carried the *eae* and *hlyA* genes, neither genes that encode for the following O antigens associated to EHEC strains: O26, O45, O103, O111, O113, O121, O145, and O157. Stx toxins represent the key virulence factor for diarrheal disease caused by STEC strains (6). Stx2 toxin is more virulent than Stx1 because that toxin is responsible for causing Hemolytic Uremic Syndrome in humans (48). Similar to the current work, a study conducted in Isla Cabritos National Park (Dominican Republic) and Granada (West Indies) failed to isolate EHEC O157:H7 from *I. iguana* (21, 49). EHEC strains belonging to serotypes O157, O26, O145, and O111 have been isolated from cases of HUS in humans from USA and Europe (50, 51). Therefore, our data indicate that *I. iguana* kept in captivity carries Stx1-producing STEC strains but not EHEC strains.

The present study also revealed that the *E. coli* isolates were grouped mainly in Clade I-II. As such, our results agree with studies demonstrating that environmental *E. coli* strains belong to different cryptic clades (52). According to this technique, the majority of *E. coli* strains could be assigned to the phylogroups mentioned above, however, in order to analyze in deep the clonality, or not, more sophisticated molecular assays such as whole genome sequence are warranted (53).

Regarding susceptibility to antibiotics, the majority of DEC strains were non-susceptible to at least three antibiotics from the different categories assessed, including penicillins, aminoglycosides, and some of the broad-spectrum antibiotics cephalothin and norfloxacin. For all DEC's strains analyzed, the MDR phenotype was mainly observed among STEC strains (*N* = 18) (Figure 1). Studies recently published showed a similar prevalence Amezquita-Lopez et al. (54) investigated antibiotic resistance (i.e., aminoglycosides, cephalosporins, penicillins, and tetracyclines) among STEC strains recovered from healthy farm animals at North Western Mexico and showed that nearly half of those strains exhibited a MDR profile; strains were associated to human disease. A study of *E. coli* strains (*N* = 841) isolated from feces of healthy cattle from northwest and western Mexico, showed that STEC and ETEC pathotypes were the most prevalent (40.7 and 26.7%, respectively); in some STEC strains (25.7%), resistance to penicillins, cephalosporins, tetracycline, and trimethoprim/sulfamethoxazole was observed (11). Another work conducted in Brazil showed that 19/57 *E. coli* strains isolated from sheep were non-susceptible to sulfonamides (57.9%), followed by non-susceptibility to ampicillin as well as cephalosporins (47.3% each); among those strains the acquired antimicrobial resistance, the gene encoding beta-lactamases was the most prevalent (55). These data, along with those described in the current study, are worrisome since treatment failure is expected in the event of a zoonosis leading to outbreaks of gastrointestinal illness. While DEC strains had not been isolated from iguanas until this study, in other studies where normal flora *E. coli* were isolated from iguanas captured at a Caribbean island, authors reported susceptibility to different antibiotics of their iguana's *E. coli* isolates (21). The non-susceptibility to antibiotics observed in DEC strains isolated from iguanas in Chiapas might indicate that resistance to antibiotics is a consequence of the acquisition of strains from humans since these reptiles are not treated with antibiotics during their life-span. Moreover, antibiotic resistance is not a common trait in bacteria isolated from wildlife animals (19). Therefore, our findings suggest a microbiological impact caused by the conditions of captivity.

The green iguana is utilized as a pet in some Latin American countries, including in South Mexico. Animals used with this purpose are considered as a potential source of zoonosis and, as observed in this study, they may also bear strains with resistance to commonly used antibiotics that may complicate infections in the event of outbreaks of zoonotic origin.

## Conclusions

The current study demonstrated the presence of diarrheagenic *E. coli* (mainly STEC) in *I. iguana* kept in captivity. The findings of the current work might be extremely useful for the sanitary efforts that are made during the green iguana handling, with the purpose of reducing its potential as a carrier for pathogenic bacteria. Additional studies about the genetic lineage of the DEC pathotypes found in the green iguana will allow us to understand the clonal origin and predict possible bacterial outbreaks in humans with DEC strains from reptiles kept in captivity, which may represent a risk for public health.

## Data Availability Statement

The datasets generated for this study are available on request to the corresponding author.

## Ethics Statement

This animal study was reviewed and approved by Committee for Animal Care of the University Autonomous of the Chiapas state (approval ID number 06/VET/RPR/269/16).

## Author Contributions

GB-T, FG-M, and BR-S conceived and design of the study. LM-G, MO-L, CI-M, PM-N, and CT-C obtained fecal samples from iguanas, as well laboratory work. LP-V and JP-B determine virulence genes and phylogenetic analyses. GB-T, JV, and JG-J performed the statistical analysis and wrote the first draft of the manuscript. All authors contributed to manuscript revision, read, and approved the submitted version.

### Conflict of Interest

The authors declare that the research was conducted in the absence of any commercial or financial relationships that could be construed as a potential conflict of interest.
